# Weighting Mean and Variability during Confidence Judgments

**DOI:** 10.1371/journal.pone.0120870

**Published:** 2015-03-20

**Authors:** Vincent de Gardelle, Pascal Mamassian

**Affiliations:** 1 Laboratoire de Psychologie de la Perception, CNRS & Université Paris Descartes, Paris, France; 2 Centre d’Economie de la Sorbonne, CNRS & Université Paris 1, Paris, France; 3 Paris School of Economics, Paris, France; 4 Laboratoire des Systèmes Perceptifs, CNRS & Ecole Normale Supérieure, Paris, France; Centre de Neuroscience Cognitive, FRANCE

## Abstract

Humans can not only perform some visual tasks with great precision, they can also judge how good they are in these tasks. However, it remains unclear how observers produce such metacognitive evaluations, and how these evaluations might be dissociated from the performance in the visual task. Here, we hypothesized that some stimulus variables could affect confidence judgments above and beyond their impact on performance. In a motion categorization task on moving dots, we manipulated the mean and the variance of the motion directions, to obtain a low-mean low-variance condition and a high-mean high-variance condition with matched performances. Critically, in terms of confidence, observers were not indifferent between these two conditions. Observers exhibited marked preferences, which were heterogeneous across individuals, but stable within each observer when assessed one week later. Thus, confidence and performance are dissociable and observers’ confidence judgments put different weights on the stimulus variables that limit performance.

## Introduction

Some choices are easy and some are not. We seem to have a sense of this difference: sometimes we are confident and sometimes we are not. This feeling of confidence matters because it tells us whether we are ready to commit to the decision, or whether we should collect more information to avoid making a mistake. However, how we make such confidence judgments and which variables contribute to these judgments, is still unclear even in simple perceptual tasks. In this domain, confidence is usually positively correlated with choice accuracy (e.g. [1–6]), and neurophysiological data seems to support the common coding of choice probability and confidence ([7]). However, the two can also be dissociated, as evidenced in several recent studies ([8–13]).

Here, we investigate how observers make such confidence judgments during a 2-choice perceptual categorization task, where two factors can be dissociated that contribute to performance: signal strength and signal reliability ([14]). On the one hand, the stimulus to be categorized can be located far from the category boundary (on easy trials) or near to it (on difficult trials). On the other hand, the stimulus can be presented with high noise (low reliability) or low noise (high reliability). In psychophysical terms, signal strength reflects the stimulus position on the x-axis of the psychometric curve relative to the point of subjective equality, whereas signal reliability corresponds to the slope of the psychometric curve.

By manipulating simultaneously these two factors, we can assess whether confidence judgments are sensitive to the expected level of performance only, or whether it is biased in addition by signal strength or reliability, above and beyond their effects on performance (i.e. signal-to-noise ratio). For instance, an observer might notice that the noise level differs between two conditions and overreact to this difference, by reporting greater confident in the low noise condition even if performance is made identical between the two conditions.

In the present study, we used a motion categorization task on randomly moving dots, whose directions followed a circular Gaussian (i.e. von Mises) distribution of which we experimentally controlled the mean (and thereby the signal strength) and the variance (and thus the reliability). Critically, we used two levels of variability (high vs. low) and we equated these two conditions in performance by adjusting the mean direction separately for high and low levels of variability. The perceptual task was embedded in a confidence comparison procedure, in which the observer indicates after two trials of the perceptual task which one was associated with the greatest confidence ([4–6]). This procedure allowed us to ask participants to directly compare their confidence between two conditions, a high-strength low-reliability and a low-strength high-reliability, which were matched in performance.

## Methods

### Participants

Fifteen adults (age range 18–30) were recruited from the French RISC database (http://www.risc.cnrs.fr). They all reported normal or corrected vision, and were naïve with respect to the goal of the study. Three additional participants were tested but discarded from all the analyses reported here, because they showed extreme behavior (distance > 3SD from the mean), two in terms of poor perceptual performance, and one for an extreme bias in the confidence task. Participants received 20 euros after their participation in two sessions in the study.

### Ethics statement

Written informed consent was obtained from all participants before the experiment. Because the research involved negligible risks and no nominative/identifying information was collected, ethics approval was not required and no IRB was consulted before conducting the study. No health information was collected from participants other than eye sight and age.

### Stimuli and notations

On each trial, two random-dots kinematograms (RDK) were presented simultaneously within circular aperatures (radius 4°), at 5° eccentricity on the left and right side of a central fixation point. Both contained motion approximately toward the center. This arrangement was preferred over one central stimulus, in order to eliminate automatic pursuit eye-movements. Both RDK contained 50 dots (radius 0.15°) whose motion directions on each frame were drawn from a von Mises distribution, which we specify here by 2 parameters: *m*_,_ the offset of the mean motion direction relative to the horizontal motion towards the center, and *v*_,_ the variance of the distribution (equivalent to the inverse of the concentration parameter of the von Mises distribution), with both *m* and *v* experimentally controlled (see design section below). The duration of the stimulus was 200ms (12 frames at 60Hz). We smoothed visual transients at stimulus onset and offset by using a linear ramp in contrast for the two initial frames and two final frames of the stimulus. Individual dots were randomly repositioned within the circular frame every 83ms (5 frames at 60Hz). At each frame, 20% of the dots were repositioned. Motion speed was constant (4° per sec). Stimuli were presented using Matlab (Mathworks, Inc.) and Psychtoolbox ([15–16]) on a 17” monitor with resolution 1024 x 768 at 60Hz, viewed at a 57cm distance.

### Task

**Fig 1:**
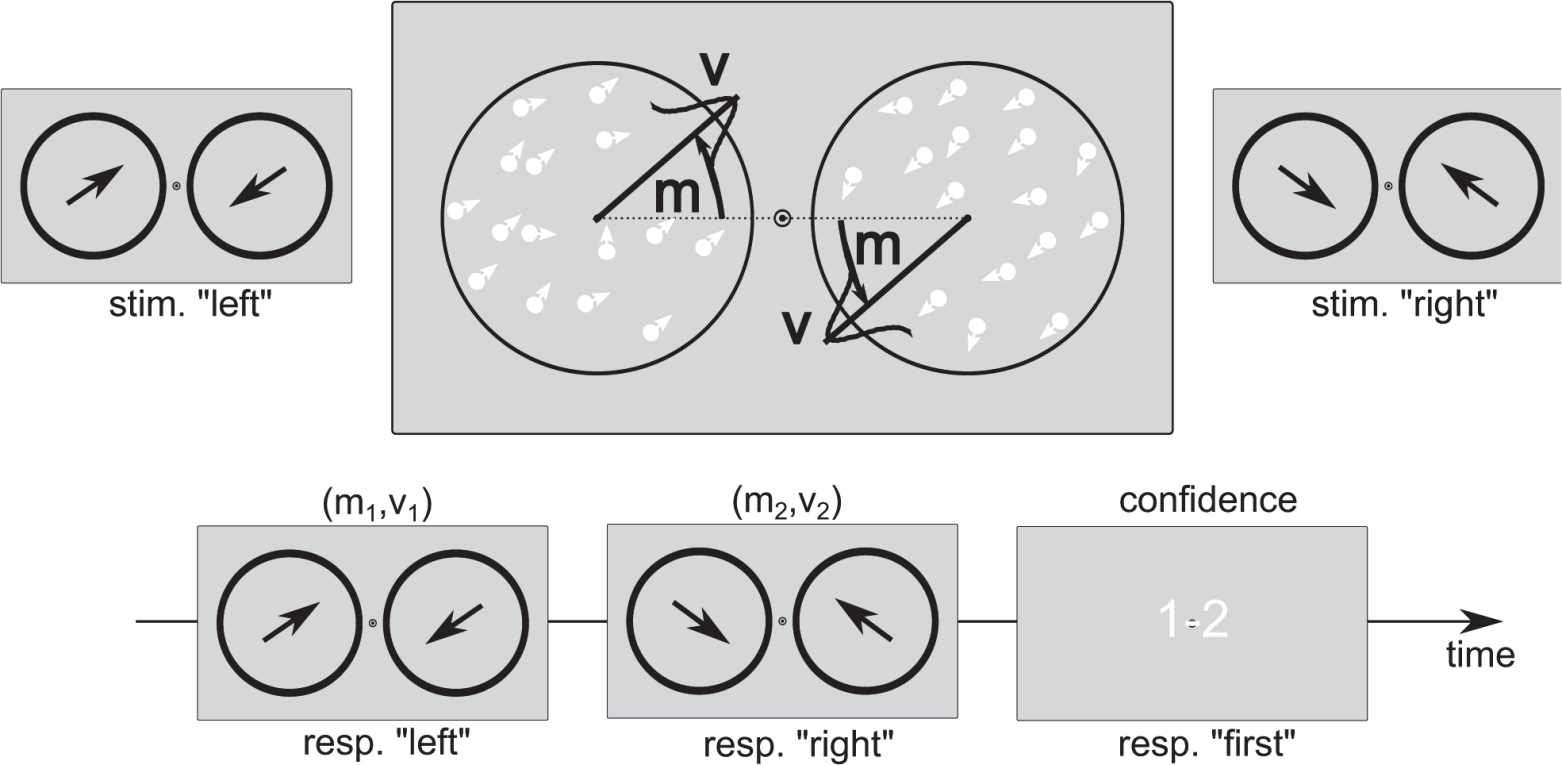
Experimental paradigm. Top: schematic representation of the stimulus and response categories. Bottom: timeline representation of a pair of first order trial, followed by a confidence comparison judgment.

There were two tasks, a first order perceptual task and a second order confidence task. For the first order perceptual task, participants had to press the left-arrow key when the left RDK moved slightly upward and the right RDK slightly downward (*m>0*), and the right-arrow key for the opposite pattern (*m<0*) (see Fig. 1). Participants were not encouraged to respond fast; their response triggered the next trial after a delay. Trial-by-trial feedback was delivered during a training phase (10 minutes), after which the confidence comparison task was introduced: after two trials, participants now had to indicate which of the two perceptual decisions was associated with greater confidence (“which was most likely to be correct”). No feedback was given during the main experiment, which lasted approximately 80 minutes.

### Design

Across trials, the variance parameter *v* was either low or high, taking values corresponding to standard deviations of 5° and 25° respectively. For each variance level, we varied the parameter *m* using staircase procedures (weighted 1-up 1-down) to maintain response probabilities for both left and right choices in the motion task at around 75% in one session and 80% in the other session. The second session occurred one week after the first session. Each pair of two trials contained one high variance and one low variance stimulus, randomly ordered, to be compared during the confidence comparison task. In each session, participants completed 560 pairs of trials.

### Analyses

**Fig 2:**
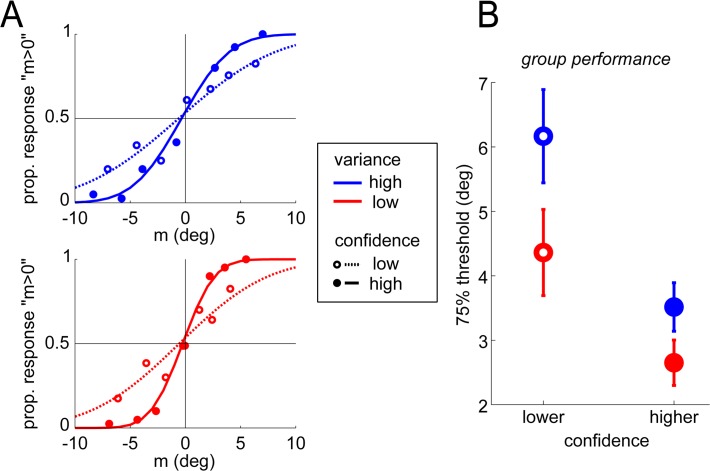
Effects of stimulus variance and reported confidence on performance. (A) Psychometric curves for one observer in one session, calculated separately for the 2 stimulus variances (red vs. blue) and the 2 confidence judgments (full vs. dotted). Dots represent binned data, lines represent fitted cumulative Gaussians. (B) Motion categorization thresholds averaged across observers, and over the two sessions, for the different conditions. Dots and error bars represent the mean and SEM of thresholds, across participants.

We quantified the motion categorization performance via psychometric curves, in which the proportion of left choices (noted PL) where described as a function of the stimulus parameter *m* (Fig. 2). The psychometric curve was fitted by a cumulative Gaussian with 2 parameters: a criterion c corresponding to the point of subjective equality between the two possible responses, and an internal noise parameter σ (EQ1). Fits were performed using maximum likelihood on the trial-by-trial data (bins in Fig. 2 are plotted only for illustrative purposes). These psychometric analyses were carried out separately for low and high stimulus variance.

$$PL\left(m\right)=\int_{x=0}^{\infty }\frac{1}{\sigma \sqrt{2\pi }}e^{-\frac{1}{2}{\left(\frac{x-\left(m-c\right)}{\sigma }\right)}^{2}}$$ (EQ1)

We report motion categorization thresholds (noted θ) as the half-distance between the two values of m for which the observer responded left in 75% of trials (PL(m) = 0.75) and right in 75% of trials (PL(m) = 0.25), on the basis of the fitted curves. In order to assess the relation between confidence and performance (Fig. 2), we also calculated the psychometric curves separately for trials associated with higher vs. lower confidence according to the confidence comparison judgment, as in previous work (e.g. [6]).

In subsequent analyses, we used this notion of threshold to quantify the signal-to-noise ratio of the stimuli, by considering for each stimulus the value *S* defined in EQ2, where *m* is the stimulus parameter, *c* is the point of subjective equality, and *θ* is the categorization threshold as defined from the psychometric curve (EQ1). Note that we obtained distinct values of c and *θ* for the two levels of stimulus variance. The quantity *S* captures both the influence of signal strength (captured by the numerator |m-c|) and signal reliability (captured by the denominator θ) on performance.

$$S=\left|\frac{m-c}{\theta }\right|$$ (EQ2)

## Results

### Perceptual performance and confidence

Motion categorization thresholds, quantifying performance in the perceptual task (see Fig. 2A), were subjected to a repeated measure analysis of variance (ANOVA) with session, stimulus variance (low vs. high), and confidence (lower vs. greater) as within participants factors. The corresponding data is presented in Fig. 2B. The analysis revealed two significant main effects. First, performance was higher (threshold was lower) for lower stimulus variance (F(1,14) = 51.46, p<0.001; mean θ: 4.84° vs. 3.51°), as expected. Second, performance increased with confidence (F(1,14) = 30.60, p<0.001; mean θ: 5.26° vs 3.08°), indicating that participants’ confidence comparison responses could capture some information about their internal noise. Additionally, a significant interaction between reported confidence and stimulus variance was found (F(1,14) = 9.81, p = 0.007), by which the variance effect was less pronounced in high confidence trials. No other effects or interactions were significant. In particular, motion categorization thresholds did not improve significantly over sessions, suggesting that participants had reached a relatively constant regime after the training in the first session.

We note that the stimuli may have differed between variance conditions (due to our staircase procedure) or confidence conditions (due to participants’ confidence being influenced by the stimuli), as reported in S1 File and S1 Fig. Crucially however, by considering the whole psychometric curve our approach allows us to estimate the effects of confidence (and of variance) on performance above and beyond these variations of the stimuli. This is illustrated by the fact that in Fig. 2A, we can see that identical stimuli (identical locations on the x-axis) were associated with better performance when participants reported higher confidence compared to lower confidence.

### Confidence biases for signal mean and variance

Our initial hypothesis was that observers would be less confident during high variance trials compared to low variance trials, even when the two trials are equalized in terms of performance (higher *v* being compensated by higher *m* parameter). To test this simply, we selected pairs of trials for which the strength of the stimuli (that is, how it deviates from the point of subjective equality, in threshold units) were roughly identical in the two trials (difference less than 1 threshold unit). We expected that in these pairs observers might be more confident in the low variance stimulus, as if stimulus variability should deteriorate confidence more than performance itself.

**Fig 3:**
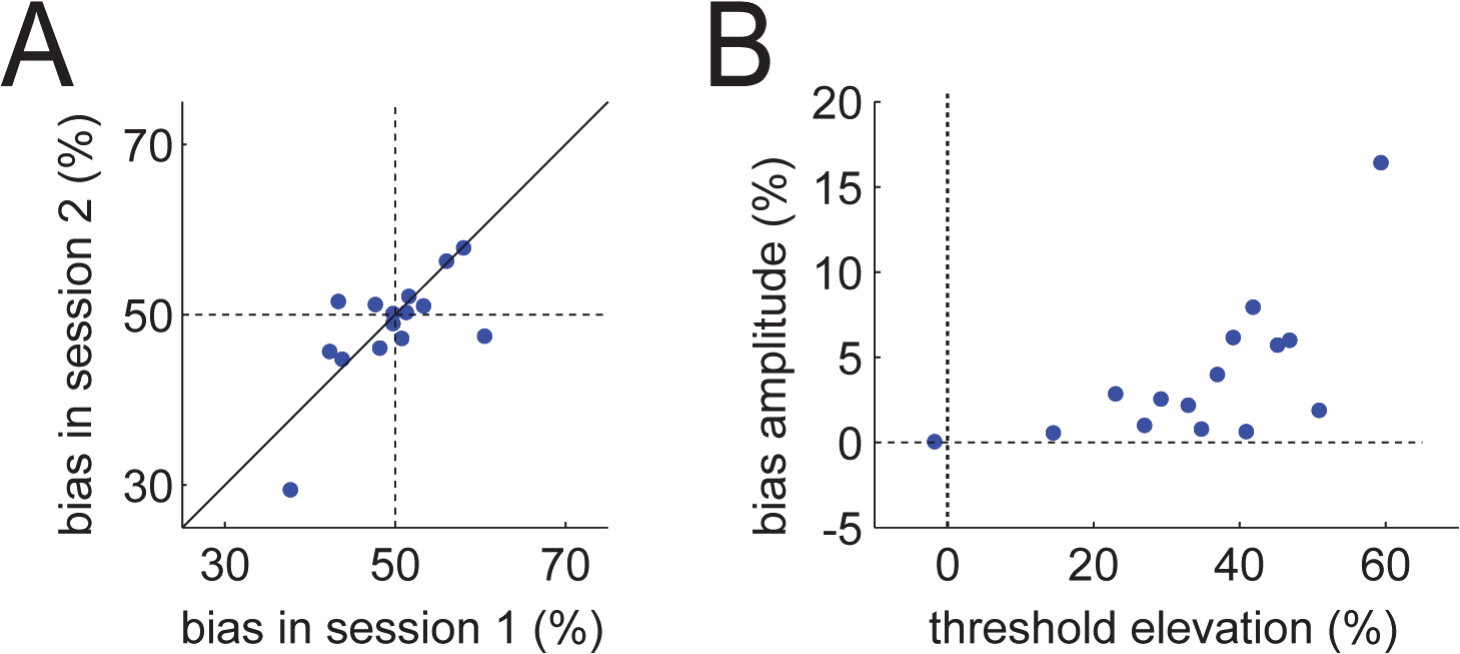
Effects of stimulus variance on confidence. In both panels each point is an individual observer. (A) The percentage of equal performance pairs in which the low variance stimulus was associated with higher confidence, calculated separately for session 1 and session 2. Individual estimates are aligned on the diagonal, showing that they were consistent across the two sessions. (B) The more the stimulus variance affected performance (x-axis: threshold elevation), the more it affected confidence (y-axis: amplitude, i.e. deviation from 50%, of the mean bias).

Overall, we found no such bias at the group level: for matched difficulty levels, some participants indicated higher confidence for the high variance stimulus while some felt more confident for low variance stimuli (Fig. 3A). Strikingly however, these individual profiles were stable and consistent over the two sessions, as indicated by a strong correlation of observers’ biases (r = 0.69, p = 0.004) between the two sessions. In other words, some observers systematically favored signals with a greater mean parameter, while others favored lower variance signals. In both cases, confidence was affected by the physical stimulus parameters above and beyond their effects on accuracy.

Two further analyses confirmed that this pattern was not driven by one participant showing heavy biases in both sessions (bottom-left datapoint of Fig. 3A). First, rerunning the correlation without the most extreme point provided almost identical results (r = 0.53, p = 0.054). Second, a robust regression analysis also confirmed a significant relation between the two biases measured in the two sessions (regression coefficient = 0.43, p = 0.03).

We then anticipated that these variance-related biases on confidence could be more pronounced in observers for whom stimulus variability had greater impact on performance. We thus calculated the amplitude of this variance bias on confidence (i.e. its distance to 50%) and the relative effect of variance on perceptual categorization thresholds (i.e. the difference in threshold between high and low variance, divided by their average), averaged across the two sessions. As anticipated, these two quantities indeed correlated across observers (r = 0.66, p = 0.007). The more variance affected performance, the more it biased confidence, in one direction or the other.

### Predicting confidence comparison using logistic regression

**Fig 4:**
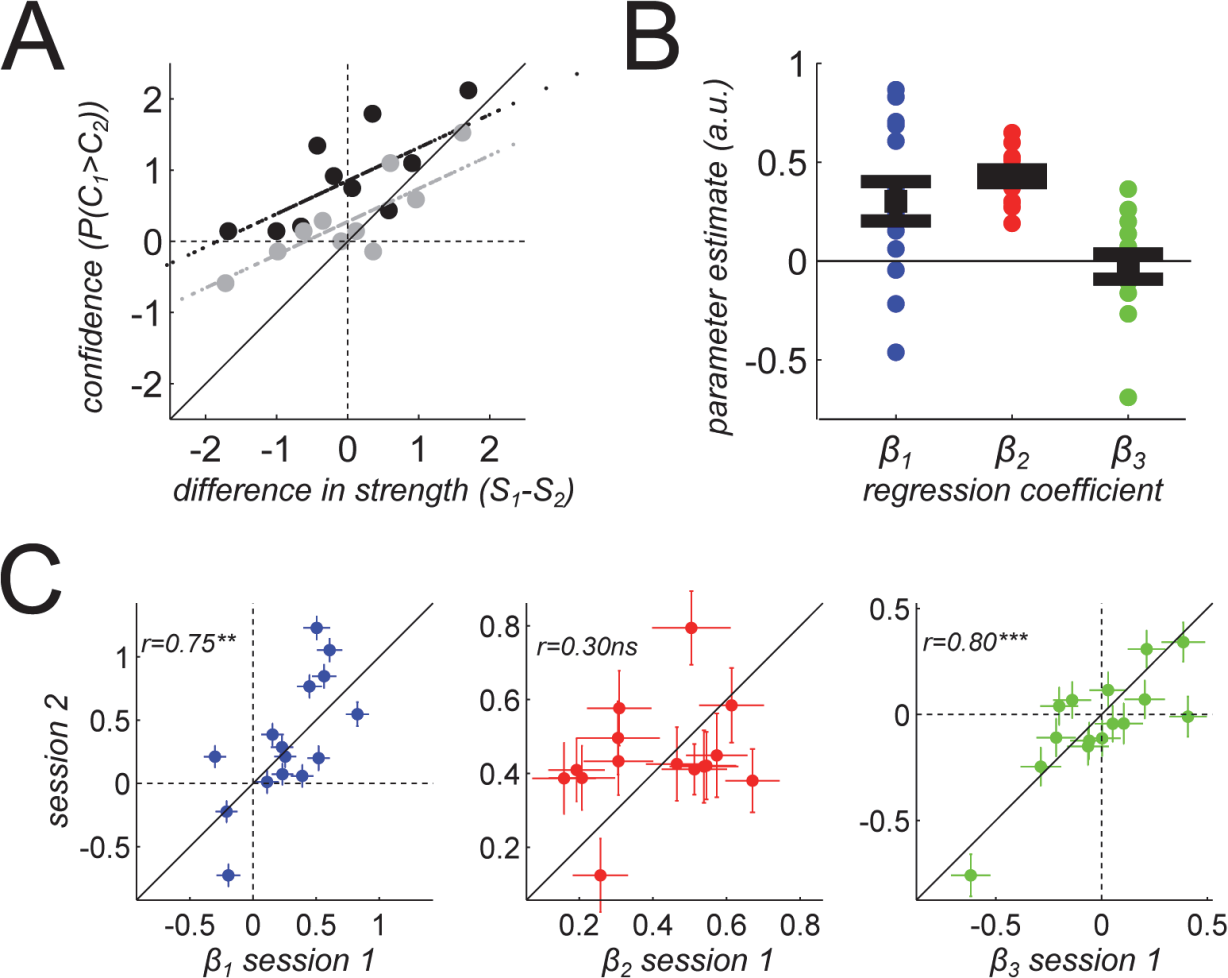
Logistic regression for confidence. (A) The metacognitive regression curve for one participant, plotting confidence comparison responses (in probability, y-axis) against differences in signal-to-noise ratios between the two trials to be compared (threshold units, x-axis). Large dots are real data, binned along the x-axis variable. Aligned small dots are regression fits. Trial pairs in which the low variance stimulus was presented first (resp. second) are presented in black (resp. gray). (B) Parameter estimates for the logistic regression (EQ1). Small dots represent for each participant the average estimate of the parameter across the two sessions. Big dots and error bars represent the mean and SEM at the group level. (C) Stability of parameter estimates across the two sessions, one week apart. For each predicting variable, we plotted the beta coefficient measured on the second session against the value on the first session, and report the correlation between the two sessions. Error bars represent the standard errors for each parameter estimate. Cross-sessions correlations are reported along with their significance (** p<0.01, *** p<0.001).

To take a different angle on these results, we carried out a logistic regression (EQ3) in which the probability that the observer reported greater confidence in the first trial of the pair, P(C_1_>C_2_), was predicted from three quantities. First, a constant term with weight *β*_1_ measured the overall tendency of the observer in this confidence comparison task. Second, we quantified using threshold units (EQ2) the signal-to-noise ratio of the stimuli in the two trials, noted S_1_ and S_2_, and we used their difference as a predictor with weight *β*_2_ in the regression. Third, to capture more formally the variance-related bias reported above, we included a predictor with weight *β*_3_, that coded whether the low variance or the high variance stimulus was presented first. This logistic regression was estimated for each participant and session. We report for each parameter the mean estimate across the two sessions, and the correlation between the two sessions to evaluate stability in time of the parameter estimates. The results of this analysis are presented Fig. 4.

$$\text{logit}\left(P\left(C_{1}>C_{2}\right)\right)=\beta_{1}+\beta_{2}\times \left(S_{1}-S_{2}\right)+\beta_{3}\times \text{sgn}\left(1/\sigma_{1}-1/\sigma_{2}\right)$$ (EQ3)

Overall, participants reported being more confident in the first trial of the pair (*β*_1_: mean = 0.30, SD = 0.38, T(14) = 3.04, p = 0.009), and this bias was stable across the two sessions (r = 0.75, p = 0.004). This bias could occur at the response or decision level, or it could indicate a real effect of time, by which observers might feel more confident in earlier trials. The regression also revealed a significant relation between confidence and signal-to-noise ratio (*β*_2_: mean = 0.43, SD = 0.12, T(14) = 13.26, p<0.001), which might constitute a measure of the metacognitive sensitivity of the observer. Of note, this sensitivity parameter did not seem to be stable in time (r = 0.30, p = 0.28). Finally, the variance-related bias exhibited a heterogeneous distribution across participants (*β*_3_: mean = −0.03, SD = 0.25, T(14) = −0.43, p = 0.67), stable across the two sessions (r = 0.80, p<0.001), which confirmed our initial results in a more formal manner. Note that, as before, this cross-session stability was also evident when the extreme datapoint was removed (r = 0.63, p = 0.015), or when a robust regression was used (regression coefficient = 0.72, p = 0.0017).

## Discussion

In the present study, we used a motion categorization task, and we manipulated two variables to limit observers’ performances: signal strength (more precisely the proximity of the signal to the category boundary) and signal reliability. In addition to the perceptual task, observers had to perform a confidence comparison task and indicate which of the last two trials was associated with the greater confidence. We report two main findings.

Firstly, we found that observers’ confidence judgments were positively correlated with performance, as previous studies have shown. Here, we obtained this positive relation in two distinct ways. A direct regression analysis showed that confidence increased with stimulus strength measured in threshold units, i.e. signal-to-noise ratio. Moreover, when we estimated categorization thresholds for higher confidence and lower confidence trials separately, we found better performance (i.e. lower categorization thresholds) when confidence was higher.

Our second result is that confidence was not based exclusively on performance or signal-to-noise ratio. Indeed, when comparing a low-mean low-variance condition and a high-mean high-variance condition that were matched in terms of performance, observers were not indifferent but they had marked preferences in terms of confidence for one condition over the other. Surprisingly, these preferences turned out to be different for different observers: some observers reported higher confidence for the “high-mean high-variance” condition and others favored the “low-variance low-mean” condition. In other words, when assessing confidence, some individuals seem to put more weight on the fact that the mean signal is far from the category boundary, while other individuals put more weight on the variability being low. These individual preferences were remarkably stable across time, and replicated in a second session one week after the first session. This result was obtained in two ways, firstly by analyzing a selection of trial pairs showing matched performance, and secondly by evaluating the bias across the whole dataset using a regression approach. This stability is important as it allows us to conclude that the individual differences we report reveal distinct individual profiles across the population, rather than random fluctuations due to sampling or estimation error.

The inter-individual variability we found in our study contrasts with a recent study by Zylberberg and colleagues ([13]) in which higher stimulus variability was systematically accompanied by lower accuracy but higher confidence. As shown previously ([9]), this counterintuitive result can be explained by a model based on signal detection theory ([17]), with the additional and critical assumption of a “unique criterion constraint” ([18]), by which the observer uses the same set of decision and confidence criteria across the two experimental conditions. In this model, the observer receives an observation sample randomly drawn from a normal distribution whose variance corresponds to internal noise, and compares this sample with a fixed set of criteria to produce a response and a confidence judgment. When the variance of the samples is greater, more samples hit extreme ratings and produce higher confidence judgments.

We note that in our study, some participants showed the same profile and reported greater confident in the higher variability trial than in the lower variability trial (at equal performance levels). Their data might be modelled along the same lines, by relying on the “unique criterion constraint” as previously shown (e.g. [9]). Critically however, other participants in our study reported lower confidence in the high variability trial than in the low variability trial, which requires further discussion. At the very least, it appears that these participants have not been limited by the “unique criterion constraint” (which is not ideal and need not be ubiquitous), and that they could adjust their criteria to the variation of internal noise.

One more general view might be proposed, according to which all participants try to adjust their decision and confidence criteria to the level of internal noise that they have to estimate in every trial. From a Bayesian perspective, this estimation of the noise variance in the current trial relies both on the current sensory information and on a prior belief about the noise variance. Thus, the inter-individual profiles we found in our study could be related to the abilities of different observers to estimate optimally the noise level. For instance, overconfidence or underconfidence in a particular condition can derive from the underestimation or the overestimation of the noise variance in the prior belief (see e.g. [19]).

In any case, the reasons why these inter-individual differences occurred in our study, but not in other studies (e.g. [9, 13]), remain to be clarified, but the stimuli or the procedure (e.g. the use of confidence ratings vs. confidence comparison) might have played a role in this issue. One could speculate that these inter-individual differences relate to the prior beliefs about the noise variance, or to the sensory sensitivity to motion variability or motion deviations. It is also possible that they relate to more cognitive phenomena like misperception of statistical variability ([20]), distortions in subjective probability weighting ([21]), or attitudes towards risk. In any case, past research has demonstrated that such individual differences do provide an interesting leverage when investigating choice behavior and metacognitive abilities, both from a behavioral and a neuroscientific perspective (e.g. [22–24]).

The question of how observers make confidence judgments has inspired several computational models in the recent years. Critically, all these models are based on the same assumption that confidence and performance rely on the exact same evidence (e.g. in Type 2 SDT [25–27]; etc.) or on the same type of evidence (e.g. [2,28]). By contrast, our work was inspired by the possibility that confidence and performance might be dissociated, and based on distinct quantities. We note that our approach is not dissimilar to cue utilization theories previously introduced in the domain of metamemory, stating that the cues used by the observer to judge confidence might be dissociated from performance in the memory task (e.g. [29–32]).

To conclude, by acknowledging explicitly that confidence might rely on other cues than performance, one opens the way for testing candidate variables that might potentially affect confidence but not performance. Delineating these variables would then inform computational efforts to understand the mechanisms of decision making and confidence judgments. We might anticipate that the set of such candidate variables is potentially very large, and contains variables related to the stimulus itself (e.g. evidence reliability, stimulus visibility, the presence of a visual mask surrounding the stimulus, etc.), variables related to the choice process (e.g. the response time, the number of alternatives, the potential rewards and stakes, etc.), variables related to the global environment in which the stimulus and response are embedded (relation with the past trials, the task load and distractions, etc.), and variables inherent to the observer (e.g. mood, stress, pain, etc.).

## Supporting Information

S1 Fig Staircase parameters. Distance between the left and right position of the staircase, averaged across trials. Dots are mean and error bars are SEM across participants. The left and right panels correspond to the ‘hard’ session and ‘easy’ session (see S1 File), respectively.
(EPS)

S1 File Staircase analyses. We report here analyses of the staircase parameters across conditions.
(DOC)
