# Supplementary material for: Weighting Mean and Variability during Confidence Judgments
Source: PLoS One. 2015 Mar 20;10(3):e0120870. doi: 10.1371/journal.pone.0120870 (PMC4368758; doi:10.1371/journal.pone.0120870)
Supplement: S1 File — We report here analyses of the staircase parameters across conditions. (DOC) [file pone.0120870.s002.doc]

**Supplementary Material for**

**Weighting mean and variability during confidence judgments**

Vincent de Gardelle^1,2,3^ and Pascal Mamassian^1,4^

(1) Laboratoire de Psychologie de la Perception,

CNRS & Université Paris Descartes, Paris, France

(2) Centre d’Economie de la Sorbonne, CNRS & Université Paris 1, Paris, France

(3) Paris School of Economics, Paris, France

(4) Laboratoire des Systèmes Perceptifs,

CNRS & Ecole Normale Supérieure, Paris, France

Corresponding author : Vincent de Gardelle, vincent.gardelle@gmail.com

Evaluation of the staircase parameters across conditions.

In the main manuscript, we report analyses of performance on the basis of psychometric curves. The data underlying these curves were obtained by sampling stimuli with different levels of the mean parameter, using staircase procedures. For completeness, we report here how the mean parameters of the stimuli differed across the different experimental conditions. Recall that the mean parameter were controlled by staircase procedures on a trial-by-trial basis, separately for each stimulus category (left vs. right), each variance condition (high vs. low), each participant and session. The point of these staircases was to obtain matched performance between the two variance conditions. Also, in one session the staircases targeted a higher level of performance (80%, the ‘easy’ session) than in the other session (75%, the ‘hard’ session).

For each participant and session, we thus computed the distance between the average values of the mean parameter in the two categories (left vs. right), for each confidence and variance combination (see Figure S1). We ran an ANOVA on this distance with session, variance and confidence as within-participant factors. As expected, we found three main effects. First, the means were further apart in the ‘easy’ session than in the ‘hard’ session (F( 1,14)= 10.84, p = 0.005), which is expected because the staircases targeted different levels of accuracy in the two sessions. Second, the means were further apart in the high variance compared to the low variance condition (F( 1,14) = 137.91, p<0.001), reflecting the higher perceptual thresholds in the high variance condition. Third, the mean parameters were further apart in the high confidence condition than in the low confidence (F( 1,14)= 55.60, p<0.001), indicating that participants’ confidence judgments were sensitive to the mean parameter of the stimulus (which of course affects perceptual accuracy). In addition, we found an interaction between variance and confidence (F( 1,14) =39.37, p<0.001), which parallels the interaction between variance and confidence found on thresholds.
